# Impact of the Transition from ICD-9-CM to ICD-10-CM on the Rates of Severe Maternal Morbidity in Arkansas: An Analysis of Claims Data

**DOI:** 10.1089/whr.2021.0092

**Published:** 2022-05-02

**Authors:** Mandana Rezaeiahari, Clare C. Brown, Mir M. Ali

**Affiliations:** ^1^ Department of Health Policy and Management, University of Arkansas for Medical Sciences, Arkansas, USA.; ^2^ Institute for Digital Health and Innovation, University of Arkansas for Medical Sciences, Arkansas, USA.

**Keywords:** ICD-9-CM, ICD-10-CM, insurance claims, severe maternal morbidity

## Abstract

**Background:**

Severe maternal morbidity (SMM) is considered as a near miss for maternal death, therefore it is crucial to identify and prevent SMM. Medical insurance claims can be used to identify SMM. There was a national transition from the International Classification of Diseases, 9th Revision, Clinical Modification (ICD-9-CM) to International Classification of Diseases, 10th Revision, Clinical Modification/Procedure Coding System (ICD-10-CM/PCS) in October 2015.

**Objective:**

This study investigates the impact of transition from ICD-9-CM to ICD-10-CM on the rates of SMM in the state of Arkansas using birth certificates linked with insurance claims data in the Arkansas All-Payer Claims Database (APCD).

**Study Design:**

Birth certificates between January 1, 2013, and December 31, 2017, were linked to insurance claims data from the APCD. SMM was defined using the algorithm provided by the Centers for Disease Control and Prevention, using ICD-9 codes for births before October 1, 2015, and ICD-10-CM codes for births on or after October 1, 2015.

**Results:**

The incidence of SMM increased after transition to the ICD-10-CM system in Arkansas. The relatively higher rate of SMM in ICD-10-CM versus ICD-9-CM was greater in magnitude on the delivery day and throughout the 42-day postpartum period (odds ratio [OR]: 1.30; 95% confidence interval [CI]: 1.20–1.42) compared with the rate on the day of delivery (OR: 1.20; 95% CI: 1.06–1.36). When excluding blood transfusions, the higher rate of SMM during the ICD-10 era was even greater both in the delivery day and 42-day postpartum period (OR: 1.66; 95% CI: 1.49–1.85) and on the day of delivery (OR: 1.58; 95% CI: 1.31–1.90).

## Introduction

Severe maternal morbidity (SMM) and maternal death are surging in the United States,^1^ with the United States being one of the only developed countries with increasing rates over recent decades. There are nearly 700 maternal deaths and an additional 5,000 SMM cases every year in the United States.^2,3^ These alarming rates of maternal mortality and morbidity call for careful identification of the rates of SMM as SMM can lead to maternal death if it is not identified and treated.

One of the challenges pertaining to understanding the trends in SMM from data that include both the International Classification of Diseases, 9th Revision, Clinical Modification (ICD-9-CM) and International Classification of Diseases, 10th Revision, Clinical Modification (ICD-10-CM) eras is the substantial change in the way hospitals conducted business as a result of transition to ICD-10-CM.^4^ Migration to ICD-10-CM has presented both challenges and benefits for the U.S. health care delivery system.^5^

The early challenges included system changes, the need for further training and education, productivity losses, and contract renegotiations.^5^ The benefits of migration to ICD-10-CM included improvement of public health surveillance data, refinement of payment systems, and improvement of fraud and abuse identification.^5^

Health care providers, payers, clearing houses, and billing services and anyone covered by the Health Insurance Portability and Accountability Act (HIPAA) were required to switch to ICD-10 codes on October 1, 2015.^6^ ICD-10-CM codes entail greater levels of detail for representation of clinical concepts. The number of clinical diagnosis codes in ICD-10-CM (69,823) is almost five times the number of diagnosis codes in ICD-9-CM (14,567).^4^ This level of difference in granularity of coding systems may misrepresent the trends of SMM (and its components) across the years.

As such, it is important to monitor these changes across years to ensure that changes in the SMM rates truly reflect the underlying health status of the maternal population. Several studies have examined the effect of transitioning to ICD-10-CM codes on numerous health outcomes. Panozzo et al. examined the prevalence of acute myocardial infarction, angioedema, ischemic stroke in patients with diabetes, and hypertension before and after the transition to ICD-10-CM codes.^7^ They found no significant difference in the incidence of AMI and hypertension across the ICD-9-CM and ICD-10-CM eras, while other outcomes did not show consistent trends.^7^

Sarayani et al. tested the performance of the existing ICD-9-CM pregnancy algorithm using the ICD-10-CM system.^8^ They found reasonable consistency in identifying the pregnancy episodes across the ICD transition period.^8^ Recently, a study investigated the impact of transition to ICD-10-CM on the incidence of SMM in the United States based on hospital discharge data. The result of this study shows reduction in the incidence of SMM by 2.26 per 1,000 deliveries as a result of transition to ICD-10-CM codes.^4^

This study provides an important landscape at the national level. We add to this important literature by evaluating the impact of the ICD-10-CM change throughout the postpartum time period, as the national data used in the previous study were limited to diagnosis codes obtained during the delivery hospitalization. Given that over half of the SMM occurs outside of the delivery hospitalization,^9^ it is critical to additionally consider how the change in ICD coding impacted SMM across the postpartum period.

In our study, SMM was identified based on having one of the Centers for Disease Control and Prevention (CDC) indicators for SMM, on the day of delivery and throughout the days postpartum (delivery day +42-day postpartum period), based on the time frame used by the World Health Organization to identify maternal mortality.^10^

Our study adds to the important literature that used hospital discharge data to identify changes in rates of SMM, by using insurance claims data to measure SMM and by including the day of delivery and 42 days postpartum.

## Materials and Methods

Data for this study came from the Arkansas All-Payer Claims Database (APCD), which is administered by the Arkansas Center for Health Improvement. The APCD contains data from multiple sources, including Medicaid and commercial insurance claims and vital statistics (*i.e.*, birth certificate) data. The APCD includes membership information and insurance claims for beneficiaries of state and federal health plans, the individual market, small and large employers, and a portion of self-insured employers.^11^

Our study included women with Medicaid coverage or private/commercial coverage, including coverage in a qualified health plan offered through the Arkansas Health Insurance Marketplace. The sample was restricted to female Arkansas residents between the ages of 12 and 55 years.

The study period spanned births occurring from January 1, 2013, to December 31, 2017. At the time of analysis, 2017 was the most recent year for which the APCD was available to be linked to birth certificates. For births that were part of a multiple birth, we included the birth certificate associated with the infant indicated as first in the birth order for the delivery. The initial sample included 148,406 birth certificates. We further excluded 14.96% of deliveries (22,199) for women who did not have medical coverage in the APCD, as indicated by missing enrollment information in the APCD overall.

Among the remaining 126,207 birth certificates, we additionally excluded 10,308 deliveries to women who did not have enrollment on the month of their delivery and 2 months after the delivery and 2,540 deliveries to women who were partially enrolled on the month of delivery and 2 months after the delivery (*i.e.*, only enrolled for 1 or 2 months within the 3-month postpartum time frame).

Next, an additional 17,773 deliveries were removed among women who had claims and enrollment information, but had information only for a secondary insurance (*i.e.*, no primary insurance indicator in the medical claims), resulting in 95,586 deliveries. Finally, we excluded 272 deliveries to women with a residence outside of Arkansas. Over 1.8 million medical claims associated with the women in our study from the day of delivery through 42 days postpartum were extracted.

SMM was identified based on the CDC algorithm that includes 16 diagnosis-based SMM indicators and 5 procedural-based SMM indicators using ICD-9-CM (January 1, 2013, to September 30, 2015) and ICD-10-CM (October 1, 2015, to December 31, 2017) codes. In addition to the SMM outcome, a second SMM measure that excluded blood transfusions was used to see the effect of change in the coding system on SMM outcomes without blood transfusions.

Several studies have investigated the rates of SMM with and without blood transfusions to account for the large percentage of SMM cases that have blood transfusion and no other indicator (∼65% of SMM cases).^12–14^ Additionally, we evaluated the effects of changes in the coding system on the rate of SMM on the day of delivery through 42 days after the delivery day (referred to as SMM_delivery day +42 days_ in the remainder of this article) as well as the rate of SMM only restricted to the day of delivery (referred to as SMM_delivery day_ in the remainder of this article).

It is important to note that SMM_delivery day +42 days_ measures SMM events that can occur during the delivery hospitalization or any time throughout the 42 days postpartum. This is important as adverse pregnancy-related outcomes can occur at the delivery or up to 1 year after the delivery.^15^ All medical claim types (*e.g.*, institutional and provider) were used to identify the SMM events in this study.

All analyses were conducted using SAS 9.4. This study was deemed nonhuman subject research by the first author's institution (#229073).

## Results

Among the total 95,314 deliveries, 56% were coded using ICD-9-CM codes. There were significant differences in characteristics of the maternal population between the two time periods (Table 1). There was a shift in maternal age, specifically a decrease in deliveries in the youngest age group, and a significant increase in maternal age from 35 to 39 in the ICD-10-CM era. There was a significant increase of births among Hispanic women after the transition to ICD-10-CM. As expected, the number of privately insured women and women with Medicaid increased in the ICD-10-CM era.

**Table 1.** Maternal Demographics of the Study Population in Arkansas in the ICD-9-CM Era (January 1, 2013, to September 30, 2015) Versus ICD-10-CM Era (October 1, 2015, to December 31, 2017)

Demographics	ICD-9-CM (Total = 53,110)	ICD-10-CM (Total = 42,204)	*p*
	***n* (% of women)**	***n* (% of women)**	
Maternal age, years			<0.0001
<20	6,933 (13.05)	5,013 (11.88)	
20–34	42,314 (79.67)	33,845 (80.19)	
35–39	3,227 (6.08)	2,825 (6.69)	
>39	636 (1.20)	521 (1.23)	
Maternal race			<0.0001
White	33,888 (63.81)	27,077 (64.16)	
Black	13,289 (25.02)	9,999 (23.69)	
Hispanic	4,007 (7.54)	3,333 (7.90)	
Other	1,926 (3.63)	1,795 (4.25)	
Payer			<0.0001
Private	10,593 (19.95)	14,885 (35.27)	
Medicaid	20,326 (38.27)	24,296 (57.57)	
Other	22,191 (41.78)	3,023 (7.16)	
Education			<0.0001
Less than high school	18,012 (33.91)	7,011 (16.61)	
High school	12,329 (23.21)	16,418 (38.9)	
At least some college	22,313 (42.01)	18,549 (43.95)	
Unknown education	456 (0.86)	226 (0.54)	

ICD-9-CM, International Classification of Diseases, 9th Revision, Clinical Modification; ICD-10-CM, International Classification of Diseases, 10th Revision, Clinical Modification.

The overall rates of SMM_delivery day +42 days_ and SMM_delivery_ during the ICD-10-CM era were 264.67 and 123.21 per 10,000 deliveries, respectively. The overall rates of SMM_delivery day +42 days_ (odds ratio [OR]: 1.3; 95% CI: 1.20–1.42) and SMM_delivery_ (OR: 1.20; 95% CI: 1.06–1.36) were significantly higher during the ICD-10-CM era relative to the ICD-9-CM era (Table 2). The rates of eclampsia, puerperal cerebrovascular disorders, and pulmonary edema/acute heart failure increased significantly following the coding change for both SMM_delivery day +42 days_ and SMM_delivery_ (Table 2). The incidence rate of disseminated intravascular coagulation was significantly lower following the coding change (Table 2).

**Table 2.** Rate of SMM per 10,000 Deliveries in Arkansas Stratified by ICD-9-CM (January 1, 2013, to September 30, 2015) and ICD-10-CM (October 1, 2015, to December 31, 2017) Time Frames and Delivery Day Versus Delivery Day +42-Day Postpartum Period

Condition^a^	SMM_delivery day +42 days_					SMM_delivery_				
	ICD-9-CM		ICD-10-CM			ICD-9-CM		ICD-10-CM		
	No. of SMM cases	Rate (95% CI)	No. of SMM cases	Rate (95% CI)	OR^b^ (95% CI)	No. of SMM cases	Rate (95% CI)	No. of SMM cases	Rate (95% CI)	OR^b^ (95% CI)
SMM	1,084	204.11 (192.13–216.63)	1,117	264.67 (249.37–280.66)	1.30 (1.20–1.42)	546	102.81 (94.36–111.8)	520	123.21 (112.85–134.27)	1.20 (1.06–1.36)
Acute myocardial infarction	—^c^	—	14	3.32 (1.81–5.57)	—	—	—	—	—	—
Aneurysm	—		—		—	—		—		—
Acute renal failure	39	7.34 (5.22–10.04)	48	11.37 (8.39–15.08)	1.55 (1.02–2.36)	14	2.64 (1.44–4.42)	12	2.84 (1.47–4.97)	1.07 (0.50–2.33)
Adult respiratory distress syndrome	68	12.8 (9.94–16.23)	65	15.4 (11.89–19.63)	1.20 (0.86–1.69)	26	4.9 (3.2–7.17)	26	6.16 (4.02–9.03)	1.26 (0.73–2.17)
Disseminated intravascular coagulation	98	18.45 (14.98–22.49)	56	13.27 (10.02–17.23)	0.72 (0.52–0.99)	60	11.3 (8.62–14.54)	31	7.35 (4.99–10.43)	0.65 (0.42–1.00)
Eclampsia	112	21.09 (17.36–25.38)	170	40.28 (34.45–46.81)	1.91 (1.51–2.43)	56	10.54 (7.96–13.69)	81	19.19 (15.24–23.86)	1.82 (1.30–2.56)
Puerperal cerebrovascular disorders	50	9.41 (6.99–12.41)	102	24.17 (19.71–29.34)	2.57 (1.83–3.61)	11	2.07 (1.03–3.71)	57	13.51 (10.23–17.5)	6.53 (3.42–12.45)
Pulmonary edema/acute heart failure	131	24.67 (20.62–29.27)	145	34.36 (28.99–40.43)	1.39 (1.01–1.77)	15	2.82 (1.58–4.66)	24	5.69 (3.64–8.46)	2.01 (1.06–3.84)
Sepsis	78	14.69 (11.61–18.33)	175	41.47 (35.55–48.08)	2.83 (2.17–3.70)	—		—	—	—
Shock	27	5.08 (3.35–7.4)	25	5.92 (3.83–8.74)	1.17 (0.68–2.01)	12	2.26 (1.17–3.95)	12	2.84 (1.47–4.97)	1.26 (0.56–2.80)
Sickle cell disease with crisis	14	2.64 (1.44–4.42)	15	3.55 (1.99–5.86)	1.34 (0.65–2.79)	—	—	—		—
Air and thrombotic embolism	59	11.11 (8.46–14.33)	72	17.06 (13.35–21.48)	1.53 (1.09–2.17)	—	—	12	2.84 (1.47–4.97)	—
Blood product transfusion	557	104.88 (96.35–113.96)	427	101.18 (91.81–111.24)	0.96 (0.85–1.09)	355	66.84 (60.07–74.17)	280	66.34 (58.8–74.59)	0.99 (0.84–1.16)
Hysterectomy	20	3.77 (2.3–5.82)	18	4.27 (2.53–6.74)	1.13 (0.6–2.14)	14	2.64 (1.44–4.42)	13	3.08 (1.64–5.27)	1.16 (0.55–2.48)

^a^ Amniotic fluid embolism, cardiac arrest/ventricular fibrillation, conversion of cardiac rhythm, heart failure/arrest during surgery or procedure, severe anesthesia complications, temporary tracheostomy, and ventilation are suppressed due to frequency less than 10.

^b^ OR of SMM in the ICD-10-CM era relative to the ICD-9-CM era.

^c^ Cells with frequency less than 10 are not shown.

CI, confidence interval; OR, odds ratio; SMM, severe maternal morbidity.

However, no significant differences were observed in the incidence rates of adult respiratory distress syndrome, shock, blood transfusions, and hysterectomy after the transition to the ICD-10-CM coding system (Table 2). Acute renal failure, sepsis, sickle cell disease with crisis, and air and thrombotic embolism had higher rates in the delivery day and 42-day postpartum period compared with the day of delivery (Table 2). Acute renal failure, air and thrombotic embolism, and sepsis had significantly higher incidence rates following the transition to ICD-10-CM.

Blood transfusions account for a large percent of SMM incidents on both the delivery day and delivery day +42-day postpartum period, as indicated in Table 2. When blood transfusions were excluded from case definitions of SMM, the overall rate of SMM remained significantly higher after transition to the ICD-10-CM system. The OR of SMM in the ICD-10 versus ICD-9 era increased after excluding blood transfusions both on the day of delivery and the delivery day +42-day postpartum period (Table 3). When adjusting for underlying demographic characteristics, the odds of SMM (with and without blood transfusions) were still higher in the ICD-10-CM era relative to the ICD-9-CM era (Table 3).

**Table 3.** Rate of SMM With/Without Blood Transfusions per 10,000 Deliveries, Stratified by ICD-9-CM (January 1, 2013, to September 30, 2015) and ICD-10-CM (October 1, 2015, to December 31, 2017) Time Frames and Delivery Day Versus Delivery Day +42-Day Postpartum Period in Arkansas

Blood transfusions	SMM timing	ICD-9-CM	ICD-10-CM	OR^a^ (95% CI)	Adjusted OR^b^ (95% CI)
		Rate of SMM	Rate of SMM		
Excluding blood transfusions	SMM_delivery day +42 days_	108.08	177.95	1.66 (1.49–1.85)	1.66 (1.47–1.87)
	SMM_delivery_	38.79	61.13	1.58 (1.31–1.90)	1.56 (1.27–1.91)
Including blood transfusions	SMM_delivery day +42 days_	204.11	264.67	1.30 (1.20–1.42)	1.30 (1.18–1.42)
	SMM_delivery_	102.81	123.21	1.20 (1.06–1.36)	1.19 (1.04–1.36)

^a^ Unadjusted OR of SMM in the ICD-10-CM era relative to the ICD-9-CM era.

^b^ Adjusted OR of SMM in the ICD-10-CM era relative to the ICD-9-CM era (adjusted for maternal demographics in Table 1).

## Discussion

Identification of trends in the incidence of SMM is important at the state and national levels. At the national level, identification of trends in SMM is important for epidemiologists and health services researchers when conducting analyses with data that span the coding system transition period. As such, continued measurement of changes in individual indicators will be critical for surveillance across populations.

According to the America's Health Rankings Health of Women and Children Report 2019,^16^ Arkansas ranked 46th of the 50 states in maternal mortality. Additionally, SMM and maternal mortality are increasing notably in rural areas,^1^ and nearly 40% of the population in Arkansas is rural with a poverty rate of 18.6%.^17^ Furthermore, previous studies have found that different populations, such as women of different races/ethnicities, have differing rates for each indicator of SMM.^18^

Our study found increases in the rates of SMM overall and SMM excluding blood transfusions after the transition to ICD-10-CM. These increases were similar when considering SMM on the day of delivery as well as on the day of delivery and throughout the 42 days postpartum. Understanding the impact of changes in the ICD coding system may provide important information for identifying trends over time and across populations.

In our study, we found changes in a number of demographic variables in the ICD-10-CM era. For example, we found increases in the number of privately insured women and women with Medicaid and a significant increase in maternal age. Arkansas expanded Medicaid under the Affordable Care Act on January 2014, commonly referred to as the “private option.”^19^

Arkansas used federal funds to purchase qualified health plans available in the Health Insurance Marketplace to expand health care coverage to low-income adults earning up to 138% of the federal poverty level.^19^ Increases were anticipated for both Medicaid and privately covered women. While most women who qualify for Medicaid coverage were enrolled into a qualified health plan, individuals who are deemed to have exceptional health care needs are placed into the traditional Medicaid program.^19^

This study has several strengths. First, we used birth certificate data to identify births rather than using a claims-based approach that relies on ICD-9-CM or ICD-10-CM codes. This approach allowed us to avoid any impact on identifying births that may occur with the coding system transition.

Second, to the best of the authors' knowledge, no study has used insurance claims to evaluate changes in the incidence rates of SMM, overall and by indicator type, following the transition to ICD-10-CM. Additionally, the use of insurance claims enabled us to investigate the intersection of SMM incidents and timing of incidents on the day of delivery versus delivery day and 42 days postpartum.

This study also has limitations. First, around 35% of the birth certificates (2013–2017) were excluded due to lack of insurance information in the APCD overall or throughout the postpartum period or because primary insurance coverage information was missing. We compared demographic characteristics among the excluded women (35,047) and the included women (95,586) and found similar characteristics in terms of age, race, and educational attainment (results available upon request).

Second, the ICD-10 codes for SMM have not yet been validated in the United States, while the ICD-9-CM codes for SMM have been validated.^20^ As such, future studies should consider evaluating differences in SMM rates after any further modifications are made to the ICD-10 algorithm.

Third, there are several variables that may strengthen the study, such as the number of units of blood products. As suggested by the American College of Obstetricians and Gynecologists and the Society for Maternal–Fetal Medicine, transfusion of four or more units of blood is one of the criteria to screen the patient for SMM^21^; however, we utilized the CDC algorithm definition for SMM of having any indicator of a blood transfusion.

Future studies should evaluate the impact of the ICD-9 to ICD-10 period in terms of the number of blood units provided.

## Conclusions

The incidence rates of SMM increased significantly after transition to ICD-10-CM in Arkansas. Exclusion of blood transfusions from the definition of SMM did not change the upward trend in incidence rates of SMM in Arkansas following the transition.

Similar to ICD-9-CM codes for SMM that have been validated in the United States, it is essential to validate the ICD-10-CM codes for SMM to allow for appropriate surveillance of SMM in the United States.

## Abbreviations Used

APCD: All-Payer Claims Database
CDC: Centers for Disease Control and Prevention
CI: confidence interval
HIPAA: Health Insurance Portability and Accountability Act
ICD-9-CM: International Classification of Diseases, 9th Revision, Clinical Modification
ICD-10-CM: International Classification of Diseases, 10th Revision, Clinical Modification
OR: odds ratio
SMM: severe maternal morbidity
